# The Impact of an Intern's Clinical Guidebook on Easing the Transition of New Interns Into the United States Healthcare System

**DOI:** 10.7759/cureus.54874

**Published:** 2024-02-25

**Authors:** Andranik Bedross, Bekure B Siraw, Ayah Alkhidir, Eli A Zaher, Parth Patel, Ashok Kumar, Peter Bostoros, Hasan Sqour, Pardeep Kumar, Shayet Hossain Eshan

**Affiliations:** 1 Internal Medicine, Ascension Saint Joseph Hospital, Chicago, USA

**Keywords:** education, intern, residency program resources, quality improvement, internal medicine

## Abstract

This study explores the efficacy of an intern's clinical guidebook in facilitating the transition of categorical internal medicine interns into the United States healthcare system. New interns, particularly foreign medical graduates, face multifaceted challenges during their initial year of residency. The research, conducted at Ascension Saint Joseph Hospital in Chicago, employed a quasi-experimental pre-post design involving 20 interns. Participants were provided with an intern's clinical guidebook, and their knowledge was assessed through pre and post exams. Results demonstrated a statistically significant improvement in overall knowledge, with mean scores increasing from 65% to 77.37%. Subgroup analysis revealed similar improvements among both male and female interns. Data confidentiality and ethical considerations were prioritized, with participant data anonymized and stored securely. Despite limitations, this study highlights the guidebook's potential to enhance intern education and improve the quality of care provided during the crucial transition period. Further research is recommended to validate and extend these findings.

## Introduction

The transition from medical school to the first year of residency presents numerous challenges for new interns. Firstly, they often face a steep learning curve as they navigate the practical application of medical knowledge in a fast-paced clinical environment [1]. Secondly, interns must adapt to increased responsibility, including making critical decisions and managing patient care independently [2]. Lastly, the demanding workload and long working hours can lead to physical and emotional fatigue, adding to the stressors of the transition period [3]. These challenges are even more pronounced on foreign medical graduates as adapting to the cultural differences and transitioning into a new environment is an additional stressor they will have to face [4].

The purpose of this study is to evaluate the effects of an intern's clinical guidebook on the transition of new interns into the United States healthcare system. Specifically, we aim to assess the changes in knowledge among categorical internal medicine interns at Ascension Saint Joseph Hospital in Chicago, before and after reading the guidebook that covers essential procedural and clinical aspects of their intern year.

## Materials and methods

Study design

A guidebook was developed by senior residents in the hospital with the intent to provide new categorical internal medicine interns with practical insight into the inpatient medical practice. This study utilized a quasi-experimental pre-post design, in which participants' knowledge was assessed before and after reading the intern's clinical guidebook. The study was conducted over the month of July 2023.

Study population

The study population consisted of 20 categorical internal medicine interns at a mid-tier teaching community hospital internal medicine residency program in the Midwestern United States.

Recruitment

Potential participants were informed about the study through email, posters, and direct communication. Interested interns were provided with a virtual participant information sheet and a consent form to review. Those who provided the informed consent were included in the study. The interns were selected based on their availability and willingness to participate in the study.

Baseline assessment

Participants were asked to complete a pre exam consisting of multiple-choice questions related to the procedural and clinical aspects of inpatient medicine covered in the guidebook. The pre exam was administered electronically, using a secure online platform. Demographic data, including age, gender, and previous medical experience, were also collected.

Intervention

Participants were given access to the intern's clinical guidebook, which was made available electronically. Participants were instructed to read the guidebook thoroughly at their own pace.

Post-intervention assessment

Following the completion of the guidebook, participants were asked to complete a post exam, which will contain similar multiple-choice questions as the pre-exam assessment. The post exam was intended to evaluate the change in knowledge gained by the participants after reading the guidebook.

Statistical analysis

Continuous variables were summarized using means and standard deviation. Categorical variables were reported as percentages. The normality of the distribution of the pre- and post-exam scores was assessed using the Shapiro-Wilk test where a p-value of >0.05 is used to define a normal distribution. Pre- and post-exam scores were compared using paired t-tests to determine the statistical significance of the knowledge improvement. Parities' pre- and post-exam score comparisons across gender were graphically represented using the ggplot2 package in R [5]. Statistical significance was set at p≤0.05. All data cleaning, visualization, and analysis were performed using R Statistical Software (v4.1.3; R Core Team 2023) [6]. 

Data confidentiality and protection

Participant data was anonymized and stored securely, with access restricted to the research team. All electronic data was stored on password-protected computers and encrypted devices.

Ethical considerations

Informed consent was obtained from all the participants before their inclusion in the study. Participants were informed of their right to withdraw from the study at any time without consequences. The study protocol was submitted to and approved by the Institutional Review Board (IRB) of Ascension Saint Joseph Hospital (approval number: RIL20230040).

## Results

Twenty participants in total participated in this project (35% being female). The participants were given the intern guidebook after taking the pre test, which included 19 questions. Thereafter, all participants were instructed to take the same 19 questions after the completion of the guidebook. The participants had a mean score of 65% during the pre intervention, while the post-intervention findings showed improved outcomes with a mean score of 77.37% (Table 1). 

**Table 1:** Descriptive statistics IQR: interquartile range; SD: standard deviation; CI: confidence interval

	Mean	Median	IQR	SD	Mean 95% CI	Median 95% CI
Post-test score (%)	77.37	78.95	15.79	12.56	71.5-83.2	57.9-97.5
Pre-test score (%)	65.00	62.50	20.00	10.76	60-70	52.4-85.25

**Figure 1:**
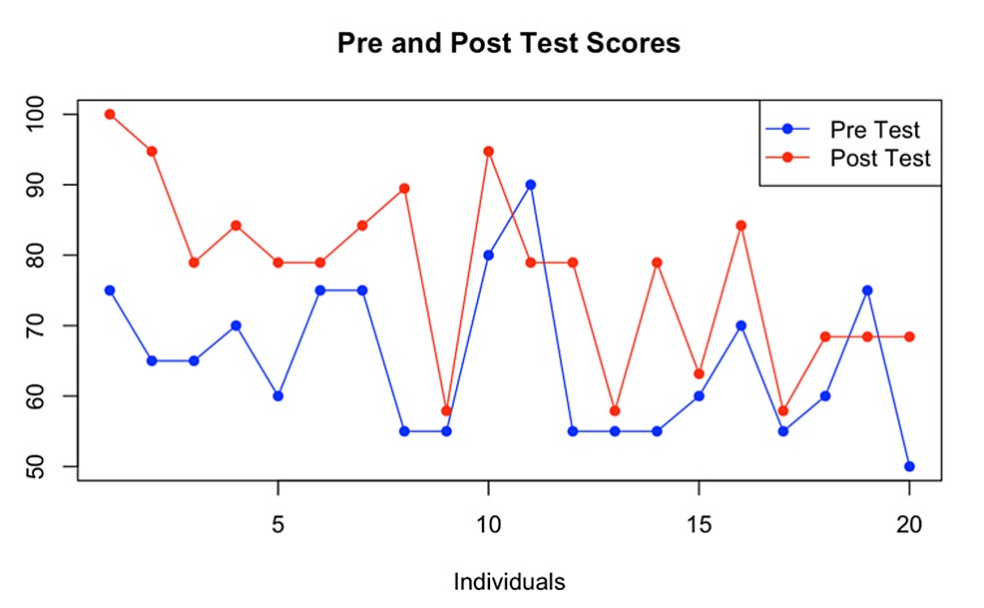
Individual scores before and after using the guidebook

The individual scores were compared and placed on a line graph (Figure 1). 

The Shapiro-Wilk test was checked and showed a pre-test score of W of 0.91306 with a p-value of 0.07091. The post-test score showed a W of 0.93932 with a p-value of 0.2328. A paired t-test was performed for the analysis of the results. The t-value was -4.6653, the degree of freedom (DF) was 19, and the p-value was 0.0001686. The confidence interval (CI) ranged from -17.917391 to -6.819451. Another paired t-test was performed comparing males and females. Males had a t-value of -3.3445 with a DF of 12, and the p-value was 0.005839. The CI ranged from -16.414251 to -3.464291. The female group had a t-value of -3.338 with a DF of 6. The p-value was 0.01565, with a CI ranging from -29.253411 to -4.505988.

**Figure 2:**
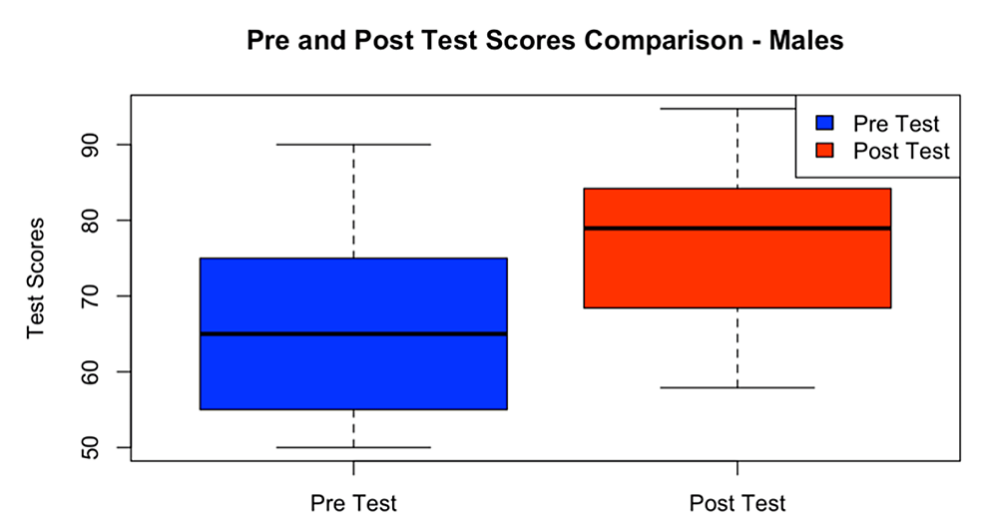
Pre- and post-test scores in male participants

**Figure 3:**
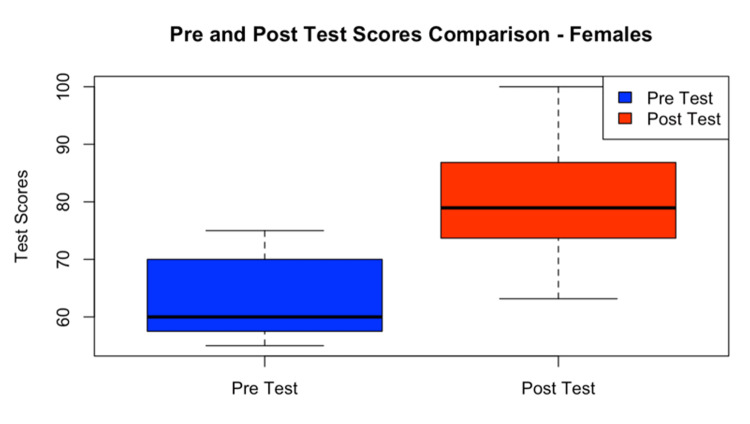
Pre- and post-test scores in female participants

A comparison plot diagram showed significant improvement in both males and females in their post- and pre-test results (Figure 2 and Figure 3).

## Discussion

Quality improvement (QI) education has been mandated for resident physicians by the Accreditation Council for Graduate Medical Education (ACGME) to do QI projects and improve the quality of residency [7]. However, QI projects have also had a bad reputation among resident physicians due to a sense of not having valuable contributions, difficulty prioritizing responsibilities related to QI projects, etc. [8]. Steps have been taken to improve QI projects by increasing awareness for QI projects, encouraging mentorship and publication, education about study design and implementation, providing books and funds, and allowing dedicated time for the project [9]. There have been multiple QI projects that have helped to improve medical management, such as decreasing sepsis-induced mortality and iatrogenic pneumothorax rates [10], improving hand hygiene in healthcare settings [11], decreasing hospital readmission rates for heart failure patients [12], improving medication reconciliation [13], implementation of early sepsis recognition and treatment protocol [14], reducing surgical site infections in orthopedic surgery [15], enhancing communication and teamwork [16], and optimizing chronic disease management in primary care [17]. It is evident that QI projects can have a significant impact on improvement in the general healthcare system.

Our study demonstrated a significant improvement in overall knowledge among categorical internal medicine interns following the implementation of the intern's clinical guidebook, with mean scores increasing from 65% to 77.37%. This highlights the effectiveness of the guidebook in facilitating the transition of new interns into the United States healthcare system and addressing the challenges they face during their initial year of residency.

Each year, medical graduates from around the world commence their internal medicine residencies in July. Typically, the orientation lectures conducted before the start of residency are overwhelming, as they are lengthy and heavy on hospital policies and healthcare legalities and often lack interactivity [18]. Many of the challenges that new residents encounter only become apparent once they begin working in the active and bustling hospital environment. Furthermore, orientation lectures often fail to effectively address these challenges, and even when they do, many residents struggle to apply the information effectively. The decline in quality of healthcare delivery and patient-related outcomes in the month of July when new doctors start residency is well studied in literature known as the "July effect" [19,20].

Significant improvement in the working knowledge of interns occurred subsequent to the introduction of an intern guidebook in our study. This improvement is credited to the direct, specific, and practical guidelines outlined in the guidebook, which was developed by residents and internal medicine attendings. The creation of such a book by peers who possess a thorough understanding of the hospital's logistics and policies can prove exceedingly beneficial in accelerating the interns' working knowledge and their ability to apply it in real-time situations.

The study will be limited to a single hospital and a small sample size, which may affect the generalizability of the findings. The self-report nature of the study may introduce response bias. The short-term follow-up limits the evaluation of long-term impacts. The study had no comparison between residents who used the guidebook and interns who did not.

## Conclusions

This study aims to investigate the impacts of an intern's clinical guidebook on the knowledge of categorical internal medicine interns during their transition into the United States healthcare system. The results showed that the intern guidebook provided statistically significant improvement in the overall knowledge of the interns. However, given some of the limitations of this study, repeat similar studies may be needed, because this study provided possible valuable insights into the effectiveness of such guidebooks in facilitating intern education and may contribute to improving the overall quality of care provided by new interns.
